# COVID‐19 in rural Africa: Food access disruptions, food insecurity and coping strategies in Kenya, Namibia, and Tanzania

**DOI:** 10.1111/agec.12709

**Published:** 2022-04-11

**Authors:** Martin Paul Jr. Tabe‐Ojong, Bisrat Haile Gebrekidan, Emmanuel Nshakira‐Rukundo, Jan Börner, Thomas Heckelei

**Affiliations:** ^1^ International Food Policy Research Institute (IFPRI) Cairo Egypt; ^2^ Institute for Food and Resource Economics University of Bonn Bonn Germany; ^3^ Apata Insights Kampala Uganda; ^4^ Deutsches Institut für Entwicklungspolitik (DIE)/ German Development Institute Bonn Germany; ^5^ Center for Development Research The University of Bonn Bonn Germany

**Keywords:** COVID‐19, food access, Kenya, Namibia, rural households, Tanzania

## Abstract

This study assesses the extent of COVID‐19‐related food insecurity in Kenya, Tanzania, and Namibia. Using the Household Food Insecurity Access Scale, we measure food insecurity in various dimensions and document several food access disruptions associated with the COVID‐19 pandemic between April and July 2020. Furthermore, we assess the association of COVID‐19 countermeasures with the adoption of various strategies in line with the coping strategies index. We rely on a unique phone survey that followed households who participated in an earlier field‐based survey. First, through Ordinary Least‐Squares and Probit regressions, we show a strong and statistically significant association between COVID‐19 countermeasures and food access disruptions and food insecurity in each of the three countries. We then use a multivariate probit regression model to understand the use of the various coping strategies, including reducing food intake, increasing food search, and relying more on less nutritious foods. We provide evidence on the complementarities and trade‐offs in using these coping strategies. COVID‐19 and related lockdown measures coincided with a deleterious increase in food insecurity in rural Africa.

## 1 INTRODUCTION

As of August 1, 2021, 9.7 million cases and over 227,000 deaths from COVID‐19 had been recorded in Africa (Hasell et al., 2020)^1^. Vaccination rates are still low in many low‐income countries (Mathieu et al., 2021), and the pandemic will likely inflict extensive adverse effects on many economic sectors across the globe (Lawson‐Lartego & Cohen, 2020). Low‐income countries where poverty and food insecurity are highly pronounced are more likely to be more affected. Recent studies have suggested that falling living standards (Egger et al., 2021; Josephson et al., 2021), increasing poverty (Sumner et al., 2020), and general economic recession will affect low‐income countries in particular (IMF, 2020; World Bank, 2020).

As the crisis continues to unfold, many African states remain vulnerable to food insecurity. Food insecurity manifests itself through disruptions in domestic food supply chains, loss of income, and reduced remittances, which all limit the capacity to purchase food (Cardwell & Ghazalian, 2020; Hobbs, 2020; Laborde et al., 2020; Pu & Zhong, 2020; Savary et al., 2020; Tamru et al., 2020). Moreover, due to restrictions in the movement of people and goods, food producers are expected to lose large amounts of easily perishable and nutritious foods (Arouna et al., 2020; Harris et al., 2020; Heck et al., 2020; Lal, 2020). Many African countries are continuously being confronted with challenging food insecurity situations (Rahaman et al., 2020) resulting in job losses and reduced economic activities. This situation is further worsened by COVID‐19 prevention policies, such as lockdowns, that limit human movements and therefore constrain access to food.

This study has three main objectives. The first is to document the level of food access disruptions emanating from constrained markets and constrained farm activities in the rural areas of Kenya, Namibia and Tanzania. We define food access disruptions as events that can inhibit or reduce a household's access to food in a manner different from the pre‐COVID‐19 norm. In this case, the main food access disruptions that the study evaluates are increased food prices (compared to pre‐COVID‐19 prices), inability to travel to markets (due to lockdown restrictions and therefore limiting food search), and general food shortages.

The second is to assess the level of food insecurity during COVID‐19 times. We employ the Household Food Insecurity and Access Scale to capture multiple dimensions of food insecurity and develop food insecurity indices that help us to observe how much COVID‐19 is associated with food insecurity.

Finally, we assess which coping mechanisms households employ in the face of COVID‐19 related food insecurity. COVID‐19 is a covariate shock for which multiple coping mechanisms might be used. Therefore, this study highlights how households combine various coping mechanisms. We rely on a 2019 baseline survey conducted in the three countries and augment this with a follow‐up phone survey administered several months after the first COVID‐19 case was registered in each of these countries. At the time of data collection between May and July 2020, Kenya and Namibia were under their first‐wave lockdown restrictions while Tanzania had just lifted them. Using a multivariate probit model, we show the associations between COVID‐19 countermeasures and various household‐level coping strategies to food access disruptions. We further explore how various strategies complement and substitute each other.

## 2 POTENTIAL IMPACTS OF COVID‐19 ON FOOD SECURITY

The literature on COVID‐19 and food insecurity is growing^2^ (Arouna et al., 2020; Barrett, 2020; Hobbs, 2020; Laborde et al., 2020; Lawson‐Lartego & Cohen, 2020; Pu & Zhong, 2020; Savary et al., 2020). Ceballos et al. (2020) show how pre‐existing infrastructure in two Indian States mitigated market disruptions. Stock‐outs also affected online markets due to reduced farm deliveries (Mahajan & Tomar, 2020), and prices of grains became unstable although minimum support prices shielded producers from very low prices (Varshney et al., 2020). Despite government support mechanisms, such as minimum prices, some products (e.g., vegetables) still suffered price drops (Ali & Khan, 2020). COVID‐19 has disrupted not only food markets but also the overall national and international supply chains (Aday & Aday, 2020; Ayanlade & Radeny, 2020; Cao et al., 2021; Elleby et al., 2020; Mahajan & Tomar, 2020), including access to agricultural inputs, such as fertilizers and others (Ayanlade & Radeny, 2020; Nchanji et al., 2021; Pan et al., 2020; Pu & Zhong, 2020). We expect many African rural households to become more food insecure due to COVID‐19 and related lockdown measures:

Hypothesis 1: Due to COVID‐19 restrictions, net consumer rural households are exposed to more significant food access disruptions such as (1) increased higher food prices partly related to, (2) the inability of sellers to access markets, leading to (3) general food shortages in markets and households.

Related to food access disruptions, preventive measures equally created employment shocks through employment losses. Evidence from low and middle‐income countries shows overall income losses due to stay‐at‐home policies (Bottan et al., 2020; Ceballos et al., 2020; Hamadani et al., 2020; Kansiime et al., 2021; Koos et al., 2020; Mahmud & Riley, 2020). Using phone‐based surveys in Mali, Adjognon et al. (2021) reported high levels of food insecurity, especially in urban areas. In Nigeria, food insecurity and shortfalls in labor market participation were also exacerbated with COVID‐19 cases and some containment measures like lockdowns (Amare et al., 2021). Moreover, the share of food‐insecure households increased by 47% (Abay, Amare et al., 2021). In some instances, social protection programs have provided some level of protection from the adverse effects of the pandemic (Abay, Amare et al., 2021; Bottan et al., 2021; Brum & De Rosa, 2020). However, such programs usually exhibit limited coverage. Correspondingly:

Hypothesis 2: Household food insecurity is likely to be worsened by movement restrictions that limit the search for both work and food. Emergency social protection interventions are not likely to fully compensate for income losses. Households are therefore likely to worry about (1) affording food due to increased prices, (2) running out of food and being unable to stockpile, (3) reducing the number of meals or going without food for some time. In addition, those who might have reserves from current and previous seasons might sell in panic, hence exposed to unfair/low prices.

Although the literature on the effect of COVID‐19 on food security in low and middle‐income countries continues to grow, minimal research exists on how rural households are coping with this shock and how different coping mechanisms complement each other.^3^ Ruszczyk et al. (2021) explored various coping strategies that poor households in Bangladesh are utilizing. The coping mechanisms employed by rural households in Kenya, Tanzania, and Namibia may differ substantially, including across country contexts in the region. Based on what is known about coping strategies in the face of multiple coinciding shocks, we expect that several complementary coping mechanisms are employed (Ansah et al., 2020).

Hypothesis 3: a single coping mechanism might not provide sufficient protection to a household faced with a macro shock (COVID‐19) that presents itself in multiple coinciding shocks. Households instead employ multiple coping mechanisms complimentarily.

We offer insights into how the pandemic has impacted food insecurity across different geographies (semiarid pastoral communities in Baringo County, Kenya; settled agricultural communities in Kilombero valley, Tanzania, and settled semiarid communities in Zambezi region, Namibia). In our analysis, we exploit the fact that all our study countries had some exposure to locust infestation at the same time as COVID‐19 countermeasures. Therefore, we account for locust shocks as an additional factor potentially associated with a households’ food insecurity situation and related coping mechanisms. By including locust infestation and other pre‐COVID‐19 shocks, we can isolate a clear association between COVID‐19 countermeasures and households’ food insecurity.

## 3 DATA AND METHODS

### 3.1 Household survey and COVID‐19 follow‐up phone survey

**FIGURE 1:**
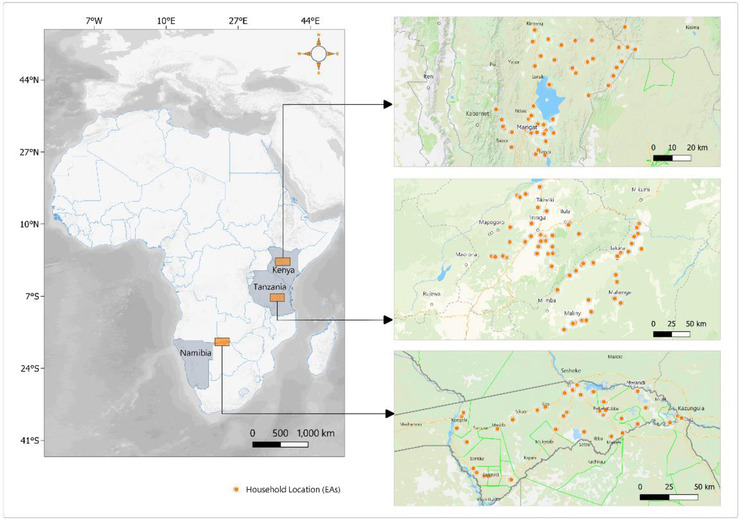
Map of study locations

Our data come from a pre‐COVID‐19 survey and a phone survey conducted in the rural areas of the Zambezi region in Namibia; Morogoro and Iringa regions in Tanzania; and Baringo County in Kenya. The phone survey followed an in‐field survey conducted between May and August 2019 under the Collaborative Research Centre–Future Rural Africa (henceforth CRC baseline). The sampling of the CRC baseline survey followed a stratified random sampling procedure, selecting 60 enumeration areas in Tanzania, 47 enumeration areas in Baringo, Kenya and 45 enumeration areas in the Zambezi, Namibia. It thus comprised 871, 652 and 704 households in Tanzania, Namibia, and Kenya, respectively. Figure 1 below shows the sample locations.

During the CRC baseline, we asked households about mobile phone ownership and recorded phone numbers. Mobile phone ownership was 70% in Kenya (494/704 households), 83% in Namibia (532/652 households), and 83% in Tanzania (723/871 households). Before the phone survey, we updated our phone ownership data through a mini phone survey with village leaders. The prospective sample was therefore increased by 14 and 166 additional households with phone numbers in Tanzania and Kenya, respectively. There were no additional households in Namibia. After updating the baseline phone records, our prospective sample was 1935 mobile phone‐owning households. In both the CRC baseline and the phone survey, we provided households with an incentive equivalent to US$ 1 of airtime. From our prospective sample of 1935 households, a response rate of 91% was achieved (1762 households). Slightly over 2% were not reached, and 6.6% did not provide consent. Compared to other COVID‐19 related phone surveys (Himelein et al., 2020), our response rates were exceedingly high.

The phone survey collected information on household socio‐demographics, food security, and health access measurements related to COVID‐19, labor markets, work‐related disruptions, and social support and assistance, among others. Critical for our purpose, information was also collected on the various measures households used to cope with food insecurity. Data were collected in June and July 2020, approximately 2 months inside local and international restrictions in Kenya and Namibia. Although Tanzania relaxed its restrictions early on and stopped recording COVID‐19 cases altogether in mid‐May 2020, our data captured the March to July timeline, partly inclusive of when restrictions were in place. However, to some extent, Tanzania provides a comparison for locations where stringent restrictions were not enforced, local markets were not extensively curtailed, and movements were not prohibited. Therefore, we expect food insecurity to be worse in other countries compared to Tanzania.

While we are convinced of our sample representativeness for the rural mobile phone‐owning households in the three study regions, some concerns may arise due to attrition. About 13% of our baseline households did not have a phone and therefore were automatically not included in the phone survey. This is contextual attrition because we do not lose these households for any other reason than phone ownership. To alleviate concerns about the possible differences between households with and without mobile phones, we use a probit regression to assess if mobile phone ownership was a significant predictor of inclusion in the phone survey using only baseline data. We establish no significant statistical relationship between phone ownership and phone survey inclusion (see supplementary materials Table A3). Thus, we are confident that our sample may not have this attrition bias. Furthermore, we follow standard measures to account for attrition by including sample selection weights that correct for any possible attrition bias (Ambel et al., 2021; Oddy et al., 2014). Our results are similar with and without including attrition weights, so the results presented include the weights.

### 3.2 Assessing COVID‐19 influence

The main variable of interest in the empirical analysis is the perception of the COVID‐19 countermeasures. In each of the three sites of the study, data collection took place when the cases were still few.^4^ Since actual disease exposure at the time was exceptionally low, we used this proxy to assess shock exposure. Other studies also used proxies such as the number of cases in the region or variation in lockdown regulations (Amare et al., 2021). Our proxy measure was the perception of the effect of COVID‐19 prevention policies on overall household welfare. We asked respondents the following question. “On a scale of 1–5, to what extent do you think COVID‐19 and related containment measures like lockdowns and curfews will affect your general life?” The Likert‐scale responses were: 1  =  very bad/negative, 2 = somewhat negative, 3 = No known effect, 4 = somewhat positive, and 5 =  very good/positive. We then created a shock exposure dummy corresponding to 1 if households expected a negative effect (responses 1 and 2), and 0 if households expected no effect or good effect (responses 3–5).

### 3.3 Outcomes

Our empirical analysis takes two dimensions. The first dimension is testing the first two hypotheses by assessing the level and correlations of COVID‐19 and (1) food access disruptions, and (2) food insecurity.

#### 3.3.1 Food access disruptions

Food access disruptions are barriers to food access that might not have existed without and are structurally correlated with COVID‐19 restrictions. We asked the following question with a yes/no response: “Have you experienced any disruptions in accessing food for your household in the last 60 days due to COVID restrictions?” For households whose response was “yes,” we inquired about food access disruptions along four dimensions, namely, (1) experience of limited availability/food shortages, increase in the price of food, inability to travel to markets, and other disruptions. These food access disruptions are likely caused by disruptions in local supply chains and market operations (Harris et al., 2020).

#### 3.3.2 Food insecurity

To measure food insecurity, we used the Household Food Insecurity Access Scale (HFIAS) (Coates et al., 2007). The HFIAS measures feelings of uncertainty or anxiety over food access (situation, resources, or supply), perceptions of food insufficiency (in both amount and quality/ nutritional diversity), and reductions in food intake. Our measures of food insecurity are also consistent with other COVID‐19‐related food insecurity studies such as Amare et al. (2021) in Nigeria. Due to the necessity to keep the phone survey short, we used three out of the nine generic questions to capture food access and anxiety. The three items from the HFIAS to measure food insecurity are (1) going without food for at least one day in the last 30 days, (2) worrying about affording food, and (3) worrying about running out of food. These three items capture dimensions of cost, availability and access. We added a fourth measure of panic selling of agricultural produce. While more attention has been allocated to panic buying food during COVID‐19 (Billore & Anisimova, 2021), it is plausible that panic selling can also happen and negatively affect rural households. When lockdowns are communicated in advance, farmers and food traders are more likely to panic sell their stock, especially perishables. They are therefore likely to take unusually low prices and low incomes. Food wastage leaves them in an even worse‐off position. We use the four questions to create a food insecurity index.

Our food insecurity index ranged from –2.4 to 1.4, with negative scores indicating more food security and food insecurity increases as the scores become positive.^5^ For comparative and robustness purposes, we develop another index – a standardized mean (score) of the four questions. The standardized mean score simply adds the dummies such that more food‐insecure households had 4 out of 4 and the nonfood insecure households had 0 out of 4. To standardize the index, we subtract the sample mean from the household score and divide it by the sample standard deviation to attain a continuous standardized index. The two indices correlate more than 95%.

#### 3.3.3 Coping mechanisms

Finally, we use the coping strategies index (Tian et al., 2008) to measure coping strategies. To keep the questionnaire lean for a phone survey, we assessed five coping strategies: (1) reducing food intake (i.e., rationing strategies); (2) increasing food search; (3) using less nutritious/ desirable food (i.e., dietary change strategies); and (4) receiving support from government; and (5) from friends and family (i.e., external help). Respondents could select multiple responses from a list of five responses in addition to “did nothing.”

### 3.4 Other control variables

Other control variables are guided by theoretical and empirical literature on strategies used by households in times of food shocks or uncertainties (Arouna et al., 2020; Barrett, 2020; Devereux et al., 2020; Laborde et al., 2020; Pakravan‐Charvadeh et al., 2020; Ragasa & Lambrecht, 2020; Savary et al., 2020; Wang et al., 2020). Some of these variables are household socioeconomic characteristics, level of social connectedness, and previous exposure to other shock events. We include household socioeconomic and demographic characteristics like age, education, household size, gender construct of the household, information access, wealth, income levels, and commercialization. Younger individuals (households) can be more proactive in using various food security strategies, their older counterparts may benefit from their large networks and build far better food resilience. Education levels improve access and use of information that can support resilience during shocks. Moreover, households may use different strategies based on the amount and accuracy of their information (Mutisya et al., 2016). We use access to internet services as a proxy for information spread.

The task of family food production has historically been gender constructed. Men have been associated with tasks such as bringing income and performing more physically demanding household activities, whereas women are more concerned with improving the food and nutrition security of most households (Tibesigwa & Visser, 2016; Theriault et al., 2017). We thus include the gender of the household head in our models to control for this relationship. In most rural areas, farm families are usually huge and made up of different members who are typically united in food consumption. Thus, we add the household size to understand how this is associated with various strategies.

In early 2020, East African countries experienced a locust infestation that was reported to be the worst in 70 years (FAO, 2020a; USAID, 2020). Although less publicized, locusts also affected Southern African countries, including Namibia (FAO, 2020b). In our sample, 16.5%, 23.7%, and 27.8% of households in Namibia, Tanzania, and Kenya, respectively, reported locust attacks on their farms. To capture the possible influence of locusts on various coping responses, we include locust attack as a control. Both locust infestation and COVID‐19 triggered emergency support programs from both governments and nongovernmental actors. But rural households would also have received support from family, friends or other individuals in their social networks. Such support might be in form of remittances and food sharing. We, therefore, included dummies for support from other actors such as governments and nongovernmental organizations and support from family and friends.

Finally, perceptions about the levels of COVID‐19 effect on households and associated coping mechanisms might depend on the pre‐COVID‐19 levels of welfare. For instance, richer households, those with more land and less exposure to shocks in previous periods, are more likely to be resilient and less food insecure. Therefore, they are less likely to perceive an adverse effect of COVID‐19 restrictions. Consequently, we include several pre‐COVID‐19 controls from the baseline survey to account for some pre‐COVID resilience levels. Finally, we account for ecological and spatial differences within and between regions by including distance to nearest towns (Nelson, 2019), human footprint (Venter et al., 2016) and area aridity that accounts for previous agricultural shocks (Trabucco & Zomer, 2019). Supplementary Table A1 provides the descriptive results and the variable descriptions.

### 3.5 Empirical specifications

To assess the association of COVID‐19 countermeasures and food access disruptions, we used a Probit model and report average marginal effects. To assess the association with food insecurity, we use OLS regressions on the continuous food insecurity index and Probit models on binary single dimensions of food insecurity.

To explore the driving factors behind utilizing and combining various coping mechanisms, a multivariate probit (MVP) estimator in the form of a conditional mixed process estimator was used (Cappellari & Jenkins, 2003). Since households used one or a combination of strategies, our empirical approach should consider the interdependency between the strategies and account for their correlations. The MVP estimator is therefore the most suitable for this assessment. Moreover, the estimator further reveals synergies, interdependences, and trade‐offs among the various strategies. It has thus been used in other food security and coping related studies (Ansah et al., 2020).

To understand the set‐up of the MVP model, consider the following: the decision to use particular food insecurity coping strategy depends on an unobservable latent variable (household's utility), which is determined by observable variables, such as age, gender, educational level, level of food disruption, social networks, food storage and availability, market access, and previous shocks faced by the household. The higher the expected utility of a strategy, the higher the likelihood that strategy will be employed. We can quantify this decision to use each strategy as a binary variable $Y_{im}$ for the latent household utility $Y_{im}^{*}$ in an MVP estimator:

(1) $$Y_{im}^{*}=\left[\delta_{m}^{\prime }X_{im}+\partial_{m}^{\prime }Z_{\mathrm{im}}+\;\varepsilon_{im}\right]\;;m\;=\;1,2,3,4,5$$

$$Y_{i}m=1\;if\;Y_{i}m^{*}>0\;\mathrm{and}\;0\;\mathrm{otherwise}$$

$\varepsilon_{im}$ m = 1,..., 5 are error terms distributed as multivariate normal, with a mean of 0 and a variance‐covariance matrix **V,** where **V** has values of 1 on the leading diagonal and correlations $\rho_{jk\;=\;}\rho_{kj}$ as off‐diagonal elements. The MVP estimator generates an error term correlation matrix that is highly informative about the interdependency between the various strategies against food insecurity. A positive correlation between the strategies’ error terms indicates complementarities or synergies while a negative correlation suggests substitutability or trade‐offs between the strategies (Cappellari & Jenkins, 2003). This is presented and discussed in the results section.

$X_{im}$ represents the COVID‐19 shock. $Z_{im}$ is a vector of control variables thought to be associated with the use of strategy *m* for household *i*, whereas $\delta_{m}^{\prime }$ and $\partial_{m}^{\prime }$ are vectors of parameters to be estimated. We used the Geweke–Hajivassiliou–Keane smooth recursive conditioning simulator where a likelihood contribution is calculated for each replication and then averaged for all the replications (Börsch‐Supan & Hajivassiliou, 1993). The simulated likelihood function is then maximized for the whole sample. This simulator utilizes a multivariate normal distribution function that can be expressed as a product of sequentially conditioned univariate normal distribution functions.

## 4 RESULTS

### 4.1 Descriptive results

First, we found that in each study area, more households expected a negative effect of COVID‐19 and associated countermeasures. Across the three countries, 72% expected a negative effect from COVID‐19 countermeasures. The negative effect was expected more in Kenya (77%) followed by 69% in Tanzania and 68% in Namibia.

#### 4.1.1 COVID‐19‐related disruptions in food access

**FIGURE 2:**
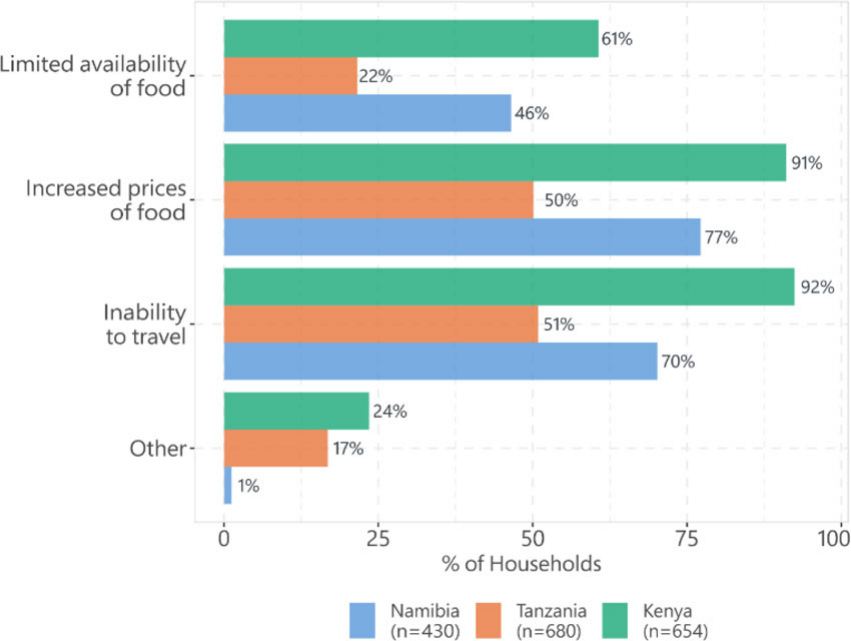
Food disruptions during COVID‐19

In the three study countries, food access disruptions are reported most in Kenya (86%) and least in Tanzania (36%). In Namibia, 74% of the households reported at least one dimension of food access disruptions. Results in Figure 2 indicate that an increase in food prices was the most reported food access disruption across the countries. In Kenya, Namibia, and Tanzania, 91%, 77%, and 50%, respectively, reported increased food prices.

The second most prominent food disruption is the inability to travel to markets due to lockdown policies. In Kenya, 92% reported an inability to travel. Meanwhile, 70% are unable to travel in Namibia and 51% in Tanzania. We also recorded “other disruptions” to food access, which are not precisely defined due to the nature of the phone survey. We show that 24% and 17% of the households experienced other disruptions in Kenya and Tanzania, respectively.

#### 4.1.2 Food insecurity during COVID‐19

**FIGURE 3:**
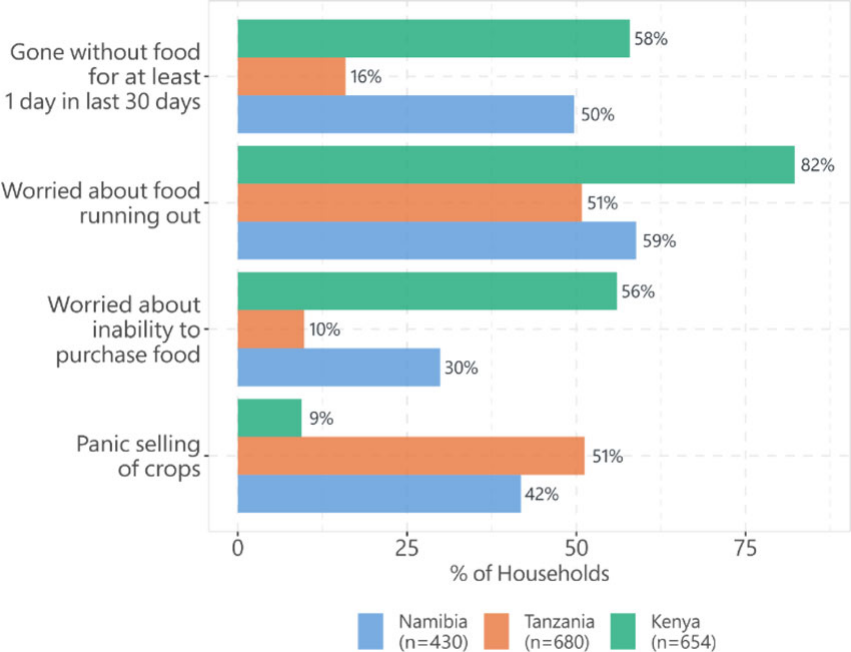
Food insecurity during COVID‐19

Food insecurity during COVID‐19 was assessed for a 30‐day recall period. Figure 3 shows the different indicators of food insecurity. In Baringo County, Kenya, we find that 58% of the households reported going without food for at least one day in the last 30 days. Going without food was lowest in Tanzania with just 16%, and about half the households in the Zambezi, Namibia, also had at least one day without food.

Worry about food security was highest in Kenya and lowest in Tanzania. Moreover, in Kenya (Namibia), 82% (59%) of the households are worried about running out of food. Meanwhile, in Tanzania, despite relaxed COVID‐19 regulations at the time of this study, 51% of the households are worried about running out of food. Moreover, 56% of households in Kenya are worried about not being able to afford food, whereas 30% and 10% are in Tanzania and Namibia, respectively. This assessment sheds some light on income losses and the limited income protection mechanisms faced by households observed in other studies (Egger et al., 2021; Josephson et al., 2021; Kansiime et al., 2021; Mahmud & Riley, 2020).

Finally, we assess panic sales of agricultural produce. The panic sale was evaluated as having sold agricultural produce that households had not planned to sell at that time. Though prevention policies such as lockdowns fueled panic buying and stockpiling of food items (Hobbs, 2020), the opposite–panic selling can also happen. With the imminent closure of markets, households are more likely to sell their easily perishable produce at unfavorable times and prices. Unfavorable sales prices coupled with high prices of imported foodstuffs might imply further exposure to food insecurity. Assessing the extent of panic selling, we find that it was highest in Tanzania as 51% of households panic‐sold some of their agricultural produce, whereas 42% did in Namibia. In Kenya, only 9% panic sold.

Considering the food insecurity index, overall, 67.6% of the sample are food insecure, corresponding with the findings of food access disruptions. More food insecurity was observed in Kenya (90.5%). Sixty‐eight per cent and 45.3% of households in Namibia and Tanzania, respectively, scored positive index values, hence food insecure. Supplementary Table A1 shows the full descriptive statistics of all control variables.

#### 4.1.3 Household coping strategies against food insecurity

**FIGURE 4:**
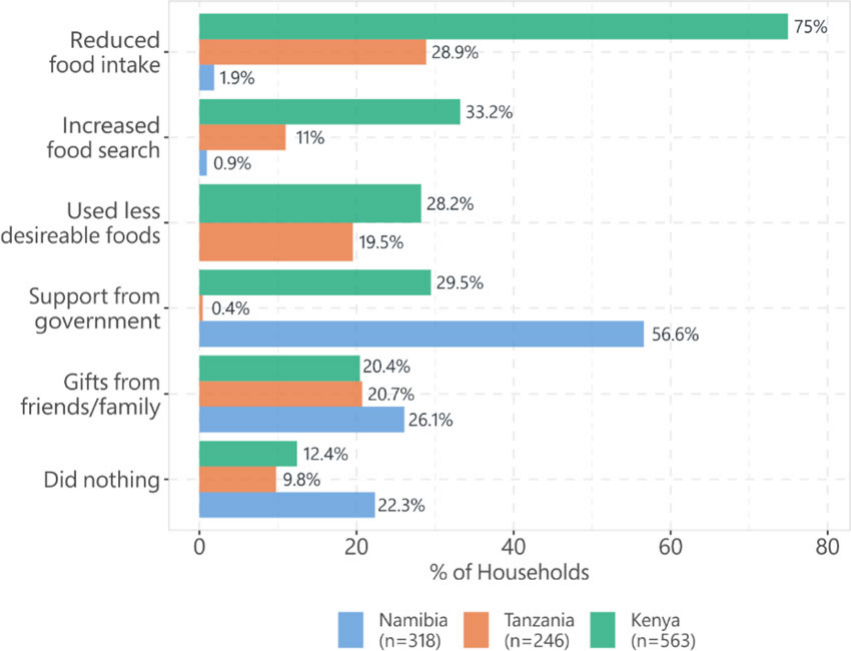
Household coping strategies

Using the coping strategies index questionnaire (Tian et al., 2008), we assessed the utilization and reliance on five critical coping strategies: (1) reducing their food intake, (2) increasing food search, (3) diversifying their food sources to include undesirable and less nutritious food, and receiving (4) food support from the government, and (5) food support from peers. Figure 4 shows the proportion of households relying on each of these strategies.

First, we observe that in Kenya and Tanzania, most households (75% and 29%, respectively) reduced food intake. In Namibia, only 2% reduced food intake, and 1% increased food search. The two main coping strategies were support from the government (56.6%) and informal support from friends and family (26.1%).

In Kenya, 33% reported increasing food search, and 28% resort to undesirable foods. Close to 30% receive some support from the government, and 20% receive in‐kind support from their friends and family social networks. In Tanzania, 20% turned to undesirable foods, and 11% increased their food search. Close to 21% reported support from family and friends, but almost no one reported any support from the government. This resonates with the policy regarding COVID‐19 response in Tanzania.

One other strategy worth noting is the “do nothing” strategy. We do not include this strategy in the regression results below. However, we note that in each of our study sites, a substantial proportion of households are faced with the COVID‐19 food insecurity shock and did not have any recourse for help. In Namibia, this proportion was the highest at over 22%. In Kenya and Tanzania, 12.4% and 9.8% did not have any support, respectively. These findings, together with support from social networks (peers), are essential for two reasons. First, they underline the covariant nature of COVID‐19 on households’ food insecurity, such that while households usually rely on their social support network during shocks (Börner et al., 2015), this strategy was less applied during COVID‐19. Second, the results reveal the limitations of formal and government‐led social support in these low‐income countries. In all sites, we observe that only a small proportion of those affected receive any help from the government. Government interventions usually come in cash or food transfers and can reduce destitution during crises (Brum & De Rosa, 2020; Gerard et al., 2020). However, if this help reaches only a small proportion of the population, the majority is left with the “do nothing” strategy, and poverty will increase.

### 4.2 Econometric regression results

#### 4.2.1 Association of COVID‐19 (counter‐measures) with food access disruptions

Table 1 shows the average marginal effects of association of COVID‐19 countermeasures with various food access disruptions. We find that COVID‐19 countermeasures are associated with increased food access disruptions in all three dimensions and the three countries. Specifically, COVID‐19 countermeasures were associated with an increase in food access disruptions of 16% in at least one of the disruptions. The countermeasures were associated with increases in food shortages of about 12%, an increase in food prices of about 15% and a 9.5% increase in food search emanating from the inability of households to travel to food markets. Across the three study areas, the association of COVID‐19 countermeasures with at least one of the three disruptions was highest in Tanzania (16.5%) and lowest in Kenya (12%).

**TABLE 1:** Association of COVID‐19 with food access disruptions

	(1)	(2)	(3)	(4)	(5)	(6)	(7)
	Any disruption	Food shortages	Food prices	Travel limitations	Any disruption (Namibia)	Any disruption (Tanzania)	Any disruption (Kenya)
COVID‐19	.159^***^	.122^***^	.152^***^	.095^***^	.157^***^	.165^***^	.120^***^
	(.030)	(.028)	(.031)	(.033)	(.060)	(.045)	(.043)
Household head is male	−.009	.037	.022	.007	−.022	−.049	.029
	(.025)	(.023)	(.027)	(.021)	(.043)	(.056)	(.030)
Secondary education	−.057^*^	−.042	−.036	−.012	.011	.015	−.095^**^
	(.031)	(.028)	(.031)	(.022)	(.055)	(.055)	(.039)
Age of the respondent	.001	.002^**^	.000	.001	.003^*^	−.001	−.000
	(.001)	(.001)	(.001)	(.001)	(.002)	(.001)	(.001)
Household size	.004	.004	−.001	−.002	.010	.014	−.004
	(.005)	(.004)	(.004)	(.004)	(.011)	(.010)	(.005)
Number of rooms	.004	−.002	−.010	.015^***^	.054^***^	−.006	.009
	(.007)	(.008)	(.007)	(.006)	(.018)	(.011)	(.011)
Household access to electricity	.028	−.018	−.017	−.058^**^	.047	.111^**^	−.022
	(.023)	(.023)	(.023)	(.023)	(.053)	(.051)	(.032)
Household access to internet	−.036	−.015	−.036	−.016	−.157^**^	.057	−.040
	(.041)	(.035)	(.041)	(.035)	(.070)	(.056)	(.055)
Crops in storage	−.048^*^	−.119^***^	−.114^***^	.026	−.094	−.064^**^	.105^*^
	(.025)	(.027)	(.026)	(.024)	(.058)	(.030)	(.059)
Locusts infestation	.008	−.035	−.028	.052^**^	.046	.108^**^	−.066
	(.031)	(.030)	(.033)	(.024)	(.069)	(.045)	(.041)
Job loss during COVID‐19	.028	.016	−.014	.031	.017	.083	.044
	(.033)	(.031)	(.033)	(.027)	(.059)	(.052)	(.037)
Membership in associations	−.003	−.000	.008	−.016	−.063^*^	−.019	.058^**^
	(.013)	(.012)	(.013)	(.014)	(.032)	(.022)	(.026)
Number of pre‐COVID‐19 shocks	.001	−.009	−.000	.006	.006	.011	−.004
	(.006)	(.006)	(.006)	(.004)	(.015)	(.012)	(.005)
Number of pre‐COVID‐19 shock responses	−.010	.004	.028	−.002	.003	−.031	−.006
	(.022)	(.018)	(.022)	(.016)	(.051)	(.048)	(.019)
Number of income sources	−.000	−.001	−.003	−.011^*^	−.022	.007	−.001
	(.007)	(.006)	(.007)	(.007)	(.015)	(.013)	(.008)
Land size	.001	.001	.001	.000	−.002	−.001	.001
	(.002)	(.001)	(.002)	(.001)	(.002)	(.007)	(.002)
Total livestock units	.000	−.000	−.000	.000	.003^*^	−.004	.001
	(.001)	(.001)	(.001)	(.001)	(.001)	(.004)	(.001)
Asset index (base: rich)							
Poorest	.025	−.016	.024	−.042	−.006	.120	.040
	(.033)	(.027)	(.035)	(.029)	(.065)	(.075)	(.049)
Poor	.026	−.021	−.002	−.025	.006	.100	.025
	(.039)	(.032)	(.043)	(.035)	(.073)	(.085)	(.045)
Average	−.001	−.034	.029	−.039	−.037	.084	.048
	(.034)	(.029)	(.035)	(.031)	(.064)	(.073)	(.036)
Non‐poor	−.019	−.005	−.040	−.034	−.080	.082	.011
	(.035)	(.031)	(.038)	(.028)	(.064)	(.095)	(.038)
Travel time to nearest town (min)	−.000	−.000	.000	−.000	.001	−.000	.000
	(.000)	(.000)	(.001)	(.000)	(.001)	(.000)	(.000)
Log aridity	.061	.033	.122	.128	1.315	−.191^*^	.626^***^
	(.112)	(.133)	(.118)	(.109)	(.865)	(.113)	(.197)
Human footprint	−.000	.005	.003	−.003	.004	−.010^*^	.010^*^
	(.005)	(.004)	(.005)	(.003)	(.008)	(.006)	(.006)
Respondent's gender is female	−.016	−.042^*^	−.043	−.021	−.033	.054	−.028
	(.029)	(.025)	(.027)	(.021)	(.053)	(.058)	(.027)
Observations	1717	1689	1742	1742	419	669	629

Standard errors clustered at enumeration area/village level in parentheses. All models include district fixed effects. Significance levels correspond with ^***^ *p* < .01 for 1%, ^**^ *p* < .05 for 5% and, ^*^ *p* < .1 for 10%.

#### 4.2.2 Association of COVID‐19 with food (in)security

Table 2 shows the association of COVID‐19 countermeasures with food insecurity. Model 1 shows the OLS regressions of the association with the overall food insecurity index and Models 2–5 show average marginal effects of the association with each of the dimensions of food insecurity measured. We find that COVID‐19 countermeasures were associated with a 36.4% increase in food insecurity. We observe that COVID‐19 countermeasures were significantly associated with all the individual measures of food insecurity. In all the measures except panic selling, there was a positive and significant association, implying that COVID‐19 was associated with worsening food insecurity. We observed a significant but negative coefficient on panic selling. This implies that having a higher perception of the negative effect of COVID‐19 countermeasures was associated with a lower likelihood of panic‐selling their agricultural produce. This would have been a strategy analogous to food stockpiling especially where access to markets would be soon limited. Models 1–3 of Table A2 in the supplementary file provides results of the association of COVID‐19 countermeasures with food insecurity indices in each country. We observed that the largest association was in Kenya. Results of the comparative standardized mean index are in Models 4 – 7 confirm these key findings.

**TABLE 2:** Association of COVID‐19 shock with food insecurity

	(1)	(2)	(3)	(4)	(5)
	Food insecurity index	going without food	food shortage (worry)	food affordability (worry)	panic selling
COVID‐19	.364^***^	.088^***^	.098^***^	.103^***^	−.040^*^
	(.108)	(.034)	(.030)	(.030)	(.022)
Household head is male	.045	.013	.025	−.001	.016
	(.075)	(.029)	(.026)	(.024)	(.020)
Secondary education	−.100	−.139^***^	.021	.008	.009
	(.088)	(.030)	(.030)	(.029)	(.023)
Age of the respondent	.003	−.000	.001	.001^*^	−.002^***^
	(.002)	(.001)	(.001)	(.001)	(.001)
Household size	−.019	−.005	−.005	−.005	.005
	(.013)	(.005)	(.004)	(.005)	(.004)
Number of rooms	.009	.000	.006	.002	.005
	(.024)	(.008)	(.007)	(.007)	(.006)
Household access to electricity	.091	−.005	.034^*^	.027	.006
	(.072)	(.027)	(.019)	(.023)	(.018)
Household access to internet	−.120	−.043	−.033	−.022	.041
	(.092)	(.037)	(.032)	(.032)	(.027)
Crops in storage	−.124	−.097^***^	−.009	−.025	.207^***^
	(.104)	(.034)	(.029)	(.025)	(.024)
Locusts infestation	.128	.028	.029	.039	.039^**^
	(.079)	(.030)	(.026)	(.028)	(.020)
Job loss during COVID‐19	.091	.082^**^	−.015	.015	.070^***^
	(.112)	(.038)	(.033)	(.035)	(.023)
Membership in associations	−.082^*^	−.007	−.023^*^	−.025^**^	.015
	(.042)	(.015)	(.013)	(.012)	(.010)
Number of pre‐COVID‐19 shocks	.015	.005	.005	.002	.004
	(.016)	(.006)	(.006)	(.006)	(.005)
Number of pre‐COVID‐19 shock responses	.061	−.000	.029	.024	.002
	(.063)	(.022)	(.021)	(.022)	(.016)
Number of income sources	.007	.003	.000	.002	.003
	(.021)	(.007)	(.006)	(.007)	(.006)
Land size	−.009^*^	.000	−.003^**^	−.003^**^	−.002
	(.005)	(.001)	(.001)	(.002)	(.002)
Total livestock units	−.004	−.001	−.002^**^	−.001	−.001
	(.003)	(.001)	(.001)	(.001)	(.001)
Asset index (base: rich)					
Poorest	.040	.081^**^	−.034	.010	.004
	(.106)	(.034)	(.033)	(.035)	(.030)
Poor	.130	.063	.023	.027	.015
	(.127)	(.042)	(.037)	(.041)	(.030)
Average	.058	.043	−.016	.038	−.002
	(.099)	(.038)	(.028)	(.034)	(.028)
Non‐poor	−.053	−.000	−.034	.010	.007
	(.095)	(.031)	(.029)	(.036)	(.023)
Travel time to nearest town (min)	.001	.000	.000	.000	−.000
	(.001)	(.000)	(.000)	(.000)	(.000)
Log aridity	−.156	.122	−.108	−.048	−.139^**^
	(.401)	(.112)	(.095)	(.099)	(.065)
Human footprint	−.010	−.006	−.001	−.002	.006^**^
	(.013)	(.005)	(.004)	(.004)	(.003)
Respondent's gender is female	.071	.012	.009	.037	−.022
	(.075)	(.028)	(.027)	(.026)	(.023)
Constant	.979				
	(3.158)				
Observations	1742	1742	1742	1717	1581
R‐squared	.278				

Standard errors clustered at village/enumeration area level in parentheses. All models include district fixed effects. Significance levels correspond with ^***^ *p* < .01 for 1%, ^**^ *p* < .05 for 5% and, ^*^ *p* < .1 for 10%.

#### 4.2.3 Association of COVID‐19 counter‐measures and food disruption coping mechanisms

This section presents the results of the MVP on the association of the COVID‐19 countermeasures and the use of various coping mechanisms.

Table 3 reports the MVP estimator results for the relationship between the COVID‐19 countermeasures and the use of different coping mechanisms by households. In Table 3, M1 corresponds with reducing food intake, M2 is increasing food search, M3 is eating less nutritious foods, M4 stands for receiving support from the government and M5 is receiving support from family, friends or other social support. We observe that a negative perception of COVID‐19 countermeasures increases the likelihood of relying on undesirable and less nutritious foods but is also strongly associated with reducing food search. This finding is expected as food search partly implies traveling to markets. When lockdown and curfew policies were in place, food search would also have been curtailed. We do not find a significant relationship between receiving support from the government and social networks of friends and family. This finding reveals two issues. The first is the covariant level of COVID‐19 shock. COVID‐19 countermeasures such as lockdowns and curfews affected households and communities in similar proportions. This implies a less likelihood of community social support being an efficient safety net. Second, we observe a negative but insignificant coefficient regarding government support during the pandemic. Given that this strategy is not utilized significantly by households, governments and other aid agencies might have also been insufficiently prepared and logistically unable to meet the food needs of households in the pandemic. Although social protection programs have shown adequate protection from the adverse effects of the pandemic (Abay, Berhane, et al., 2021; Bottan et al., 2021; Brum & De Rosa, 2020), they tend to cover only a small proportion of the population. Moreover, under a covariant shock such as COVID‐19, many vulnerable and food‐insecure households may remain uncovered by even the most comprehensive emergency support programs.

**TABLE 3:** Association of COVID‐19 shock with household's choice of coping mechanisms

	(1)	(2)	(3)	(4)	(5)
	M1	M2	M3	M4	M5
COVID‐19	.176	−.353^**^	.835^***^	−.089	.180
	(.163)	(.151)	(.212)	(.154)	(.136)
Household head is male	.126	.186	−.107	−.070	.014
	(.123)	(.146)	(.119)	(.110)	(.114)
Secondary education	−.114	−.203	.256	.180	.026
	(.146)	(.149)	(.157)	(.130)	(.130)
Age of the respondent	−.000	−.009^*^	.009^*^	−.005	.005
	(.004)	(.005)	(.005)	(.003)	(.004)
Household size	−.041^*^	.001	.044^**^	.013	−.032
	(.021)	(.021)	(.019)	(.020)	(.020)
Number of rooms	−.020	−.020	.051	−.055	.029
	(.039)	(.042)	(.042)	(.039)	(.031)
Household access to electricity	.088	−.209	.065	−.110	−.237^*^
	(.126)	(.164)	(.134)	(.161)	(.128)
Household access to internet	−.260^*^	.059	.089	−.074	.336^**^
	(.152)	(.221)	(.166)	(.223)	(.157)
Crops in storage	−.133	−.469^***^	.062	.698^***^	.215
	(.160)	(.176)	(.162)	(.169)	(.143)
Locusts infestation	−.125	.037	−.117	.499^***^	.303^**^
	(.157)	(.147)	(.160)	(.163)	(.121)
Job loss during COVID‐19	−.009	.266	.144	.277^*^	.297^**^
	(.166)	(.200)	(.160)	(.142)	(.122)
Membership in associations	−.003	−.064	−.063	.077	.017
	(.074)	(.090)	(.068)	(.077)	(.062)
Number of pre‐COVID‐19 shocks	−.008	.012	−.018	−.036	.018
	(.029)	(.031)	(.029)	(.029)	(.028)
Number of pre‐COVID‐19 shock responses	−.135	.190^*^	−.150	−.087	.151
	(.112)	(.111)	(.117)	(.092)	(.104)
Number of income sources	.038	.005	−.035	.054	.015
	(.035)	(.039)	(.041)	(.037)	(.040)
Land size	−.004	.002	−.008	.002	−.011
	(.007)	(.006)	(.008)	(.007)	(.007)
Total livestock units	−.003	−.002	−.004	.004	.002
	(.003)	(.004)	(.004)	(.003)	(.003)
Asset index (base: rich)					
Poorest	−.045	−.071	.156	.080	.027
	(.177)	(.180)	(.174)	(.182)	(.152)
Poor	.143	−.074	.136	.165	.016
	(.173)	(.241)	(.223)	(.213)	(.151)
Average	.017	.059	.004	.032	−.047
	(.160)	(.177)	(.256)	(.179)	(.139)
Non‐poor	.121	−.277^*^	.048	.139	−.125
	(.143)	(.165)	(.197)	(.169)	(.114)
Travel time to nearest town (min)	−.002	−.003^*^	.002	−.002	−.001
	(.002)	(.002)	(.002)	(.002)	(.002)
Log aridity	−.518	−.695	−.141	.553	.043
	(.387)	(.606)	(.437)	(.581)	(.511)
Human footprint	.004	−.015	.040^*^	.023	−.019
	(.022)	(.028)	(.022)	(.019)	(.015)
Respondent's gender is female	−.303^**^	−.339^**^	.138	.015	.034
	(.127)	(.139)	(.120)	(.105)	(.125)
Constant	2.393	4.240	−2.133	−4.477	−1.302
	(3.171)	(4.794)	(3.688)	(4.644)	(4.143)
Observations	1113	1113	1113	1113	1113

*Note*: M1 = Reduced food intake M2 = Food search M3 = Less nutritious foods M4 = Food support from government M5 = Food support from peers. Standard errors clustered at village/enumeration area level in parentheses. All models include district fixed effects. Significance levels correspond with ^***^ *p* < .01 for 1%, ^**^ *p* < .05 for 5% and, ^*^ *p* < .1 for 10%.

Highlighting other associations with coping strategies, we observe that households with older respondents, the majority of whom (65.3%) are household heads, are more educated, and that larger households are more likely to rely on less nutritious foods. Moreover, households with older respondents are less likely to increase food search, and larger households are less likely to reduce the overall quantity of food intake as well as increasing food search. We find that having some agricultural produce stored by the households is particularly important. It reduces a household's likelihood of increasing food search. However, crop storage is associated with receiving support from the government and friends. In this study, we cannot explain why and how such a finding suffices, but one can postulate elite capture (i.e., better‐off households may also benefit from external relief efforts) and informal barter exchange arrangements. We find that pre‐COVID‐19 income is associated with relying on reducing food intake and receiving less from informal networks. Consistent with income, as households become well off along a wealth spectrum, they are less likely to search for food but more likely to eat less nutritious foods. Moreover, they are less likely to receive support from informal networks to some extent. Finally, we find that locust shock did not increase or reduce food search, less nutritious food, or food intake in general. However, we observe increased support from governments, aid agencies, and social network support. Locusts are a less covariate shock compared to COVID‐19. Households can support each other, and governments and aid agencies can logistically target those who are most affected.

#### 4.2.4 Complementarities among coping strategies

The MVP estimator allows us to report interdependencies between various food access strategies. The MVP estimator generates correlation matrices that enable the observation of synergies and trade‐offs in using the various strategies. Table 4 shows these correlations. COVID‐19 is a large scale covariate shock on households; thus, we expect that households employ multiple coping strategies. Single strategies might be insufficient. Therefore, we expect strong correlations between the strategies. As expected, significant correlations are observed between the utilization of various mechanisms, suggesting complementary relationships.

**TABLE 4:** Correlation matrix of coping strategies

	M1	M2	M3	M4	M5
M1	1				
M2	.232^**^	1			
	(.108)				
M3	.357^***^	.023	1		
	(.091)	(.073)			
M4	.045	−.190	.051	1	
	(.101)	(.119)	(.108)		
M5	−.026	−.145	−.074	.337^***^	1
	(.077)	(.106)	(.069)	(.075)	

*Note*: M1 = Reduced food intake M2 = Food search M3 = Less nutritious foods M4 = Food support from government M5 = Food support from peers. Standard errors clustered at enumeration areas are in parentheses. Significance levels correspond with ^***^ *p* < .01 for 1%, ^**^ *p* < .05 for 5% and, ^*^ *p* < .1 for 10%.

We observe that reducing food intake is highly associated with eating less nutritious foods and increasing food search. We also observe that receiving support from family and friends is associated with receiving support from governments and aid agencies. In countries like Kenya and Tanzania, widespread mobile money services enable remittances. Moreover, governments and aid institutions also rely on similar services to reach affected households during humanitarian situations (Bailey, 2017). Therefore, these strategies tend to complement each other. The downside of this complementarity is the greater tendency for less‐connected households to be excluded from relief efforts.

#### 4.2.5 Heterogeneity in the three countries

We are further interested in highlighting the cross‐country heterogeneity. To observe heterogeneity, we conduct individual countries' analyses and report the results in Table 5. In Kenya, all five coping mechanisms are employed by households, and the results are more or less similar to the pooled model in Table 4. Moreover, households in Kenya reduced food search but increased reliance on less nutritious and undesirable foods. As in the pooled model, we also observe that households in Kenya received support from both the government and social networks with locust shocks.

**TABLE 5:** Heterogeneous assessment of coping strategies across three sites

	M1	M2	M3	M4	M5
Panel 1: Kenya					
COVID ‐19 shock	−.197	−.331**	.682***	−.086	.224
	(.223)	(.145)	(.231)	(.196)	(.250)
Observations	563	563	563	563	563
Panel 2: Tanzania					
COVID‐19 shock	.436*	.665	1.081**		.084
	(.243)	(.424)	(.440)		(.258)
Observations	241	241	241		241
Panel 3: Namibia					
COVID ‐19 shock				−.091	−.019
				(.202)	(.226)
Observations				309	309
Other controls	Yes	Yes	Yes	Yes	Yes

*Notes*: Each panel is an independent regression. M1 = Reduced food intake M2 = Food search M3 = Less nutritious foods M4 = Food support from government M5 = Food support from peers. Standard errors in parentheses adjusted for 147 clusters/enumeration areas.

*** *p* < .01.

** *p* < .05.

* *p* < .1.

All models include the full set of controls as in the pooled model in Table 3.

Table 6 presents correlation matrices by country to assess the synergies and complementarities within countries. Panel 1 shows the correlation matrix for Kenya, Panel 2 shows Tanzania and Panel 3 shows Namibia. Because not all strategies are used in all countries, some strategies drop out in Tanzania and Namibia. In addition, to allow for full convergence of the model, we omit district fixed effects from the country‐level models.

**TABLE 6:** Correlation matrices of coping strategies by country

	M1	M2	M3	M4	M5
Panel 1: Kenya					
M1	1				
M2	.503^***^	1			
	(.125)				
M3	.386^***^	.123	1		
	(.119)	(.091)			
M4	.070	−.125	.049	1	
	(.117)	(.125)	(.105)		
M5	−.050	−.104	−.008	.497^***^	1
	(.112)	(.118)	(.085)	(.120)	
Panel 2: Tanzania					
M1	1				
M2	−.527^**^	1			
	(.209)				
M3	.456^***^	−.400	1		
	(.166)	(.255)			
M5	.224^*^	−.532^**^	−.150	1	
	(.119)	(.270)	(.165)		
Panel 3: Namibia					
M1	1				
M2	.062	1			
	(.102)				

*Note*: Each panel is an independent regression. M1 = Reduced food intake M2 = Food search M3 = Less nutritious foods M4 = Food support from government M5 = Food support from peers. Standard errors clustered at enumeration areas are in parentheses. All models include the full set of controls. Significance levels correspond with ^***^ *p* < .01 for 1%, ^**^ *p* < .05 for 5% and, ^*^ *p* < .1 for 10%.

The complementarity and substitutability of coping mechanisms in Kenya very much mirror the full pooled model. Households in Kenya that reduce food intake are also more likely to increase food search and eat less favored food. However, those that receive support from the government are also more likely to receive help from family and friends.

In Tanzania, the situation contrasts somewhat with the one in Kenya. Households that reduce food intake are less likely to increase food search but instead more likely to use less preferred foods and more likely to receive support from their social support network. However, those who receive support from their network are less likely to resort to less preferred food. Having several negative correlations in Tanzania indicates that food insecurity would have been less pronounced such that the use of multiple coping mechanisms was less common. We do not observe any significant complementarity between the two coping mechanisms used in Namibia.

## 5 DISCUSSION AND CONCLUSION

This study investigated the level of food insecurity in mid‐2020 when African countries were alert to increasing COVID‐19 cases and thus implementing restrictive policies to reduce infections. Although these restrictive policies might have averted many infections, they allegedly curtailed economic activities and worsened food insecurity in many rural households.

We used a unique phone‐based survey data from 1749 smallholder households across Kenya, Tanzania, and Namibia. We then posit three hypotheses relating to (1) increased food access disruptions emanating from prevention policies. These disruptions would (2) worsen food insecurity in various dimensions. Due to the nature of COVID‐19 as a macro and covariate shock, (3) households would have to employ multiple coping strategies to deal with food insecurity. In line with our first hypothesis, descriptive results show that most households across the three countries suffered in at least one dimension of food insecurity. In all three countries, households were worried about running out of food more than about foregoing consumption in other dimensions. Moreover, a substantial proportion of households engaged in panic selling of their crop produce during COVID‐19 and many households experienced multiple food access disruptions, with Kenya being the most affected of the three countries. Probit regressions confirm these associations that households most affected by COVID‐19 countermeasures are more likely to suffer higher food access disruptions. However, while COVID‐19 countermeasures are associated with food access disruptions in Namibia, country‐level subsample analysis shows that these food access disruptions did not translate into extensive food insecurity in households.

We find corroborative evidence to support hypothesis two – that household food insecurity would likely worsen due to food access disruptions in accessing markets. Food insecurity was experienced in each of the three countries studied, especially Kenya, where 82% worried about food, 58% went for at least one day without food and 56% are worried about the inability to purchase food. Significant levels of food insecurity are also pronounced in Namibia and Tanzania. We do not find significant associations with panic selling of farm produce though a substantial proportion of households reported selling at unfavorable periods. Our work here contributes to other studies such as Hirvonen et al. (2021) who found extensive market disruptions in Ethiopia, especially affecting vegetable and other perishable producers as households reduced consumption. However, increase in vegetable consumption in Guatemala (Ceballos et al., 2021) could also be an indication of disruptions in fresh food markets.

However, we observe that Tanzania was partly insulated as it did not suffer as much food insecurity compared to Kenya and Namibia. Tanzania did not implement similar COVID‐19 countermeasures as many other countries (Buguzi, 2021). As seen in Figure 4, coping mechanisms employed also mirror prevention policies implemented. For instance, in Tanzania, no respondent received government support because the government partly downplayed COVID‐19′s existence (Buguzi, 2021). It was further argued that standard policies were untenable in the country for livelihood and food security reasons (Mfinanga et al., 2021). Our results corroborate other studies that have also found higher levels of food insecurity during COVID‐19 in other low‐income countries (Durodola et al., 2020; Nchanji & Lutomia, 2021). However, most households still sold their produce in panic in periods they would not have sold it without the COVID‐19 situation.

The association of COVID‐19 shock with food insecurity in Namibia was less pronounced and not statistically significant. We also found that households reduced food intake and increased their reliance on undesirable foods.

Regarding coping strategies, we found that reducing food intake was the most frequently mentioned strategy in Kenya and Tanzania while in Namibia, support from the government was the most frequent. In Namibia, only two coping mechanisms are predominant, and the others are dropped from the model due to extremely low reliance rates (Figure 4). We do not see any significant associations implying that even without the COVID‐19 shock, households in Namibia might have used these mechanisms for their usual consumption smoothing.

Combining the phone survey data with pre‐COVID‐19 data, we assessed associations between the COVID‐19 shock and various coping mechanisms. Further, we controlled pre‐COVID‐19 socioeconomic conditions, implemented an MVP estimator, extracted associations, and revealed complementarities between various strategies. Results from the MVP estimator show that COVID‐19 is associated with dependence on rationing (reducing food intake) and dietary change (relying on less nutritious and undesirable foods) strategies. However, government/humanitarian and informal social support systems are insufficient due to the extent of the covariate shock. In line with our third hypothesis, households apply multiple coping mechanisms with strong complementarities among strategies based on modifying food intake and relying on formal and informal support networks.

Overall, our findings suggest that rural households in Kenya, Tanzania, and Namibia have suffered food access disruption. This may be due partly to the national lockdowns and the already looming food insecurity in most rural areas. Given the extent and scope of food intake and the reliance on less nutritious foods, social and governmental support is needed and would go a long way in offsetting some of these food insecurities. Apart from institutional support, food storage and market stabilization policies and action are needed. While vaccinations continue to lessen the negative effects of COVID‐19 in many countries, policy makers will be concerned about the urban populations from whom economic involvement was largely curtailed. Although policymakers might be more concerned about urban poverty, governments need not overlook the lived realities of the marginalized rural poor who are not reached by relief efforts.

## Supporting information

Supplementary information

Supplementary information

## Footnotes

1 is the citation for a daily updating database of worldwide COVID‐19 cases and testing, which can be accessed at https://github.com/owid/covid‐19‐data/tree/master/public/data

2 For instance, a Web of Science search with the key search words “COVID‐19” AND “food insecurity OR food security” covering 2020 and 2021 up to August 2, 2021, produced 527 results.

3 A Web of Science search with the key search words “COVID‐19” AND “food insecurity OR food security” AND “coping mechanisms” covering 2020 and 2021 up to August 2, 2021, produced only three results.

4 By end of July 2020, Kenya had 20,636 cases and Namibia had 2,129 cases (Hasell et al., 2020). Meanwhile, Tanzania was not reporting any COVID‐19 data.

5 The index here is developed using principal components analysis and keeps the first principal component as the index.
