# Supplementary material for: COVID‐19 in rural Africa: Food access disruptions, food insecurity and coping strategies in Kenya, Namibia, and Tanzania
Source: Agric Econ. 2022 Apr 11;53(5):719–38. doi: 10.1111/agec.12709 (PMC9111212; doi:10.1111/agec.12709)
Supplement: Supplementary file 1 — Supplementary information [file AGEC-53-719-s002.docx]

**COVID-19 in Rural Africa: Food Access Disruptions, Food Insecurity and Coping Strategies in Kenya, Namibia, and Tanzania**

Tabe-Ojong et al.

**Appendix Tables**

Table A1 Summary statistics of control variables

|  | Kenya | | Namibia | | Tanzania | |
| --- | --- | --- | --- | --- | --- | --- |
|  | mean | sd | mean | sd | mean | sd |
| Household head is male | 0.673 | 0.47 | 0.63 | 0.482 | 0.503 | 0.5 |
| Secondary education | 0.255 | 0.436 | 0.678 | 0.468 | 0.132 | 0.339 |
| Age of the household head | 40.113 | 13.924 | 44.706 | 15.723 | 44.849 | 15.029 |
| Household size | 6.388 | 2.909 | 4.998 | 2.396 | 4.597 | 2.187 |
| Number of rooms | 2.139 | 1.346 | 2.431 | 1.334 | 3.806 | 1.811 |
| Access to electricity | 0.239 | 0.427 | 0.231 | 0.42 | 0.498 | 0.498 |
| Household has internet access | 0.131 | 0.338 | 0.082 | 0.274 | 0.099 | 0.298 |
| Crops in storage | 0.116 | 0.321 | 0.367 | 0.483 | 0.454 | 0.498 |
| Locust infestation | 0.278 | 0.448 | 0.166 | 0.372 | 0.237 | 0.425 |
| Lost work during COVID-19 | 0.176 | 0.381 | 0.206 | 0.405 | 0.134 | 0.341 |
| Membership in social support groups (number) | 0.232 | 0.599 | 0.325 | 0.608 | 0.594 | 0.898 |
| Number of pre-COVID-19 shocks (weighted) | 4.532 | 2.287 | 3.74 | 1.659 | 3.338 | 1.636 |
| Number of shock responses used pre-COVID-19 | 0.788 | 0.579 | 0.514 | 0.465 | 0.627 | 0.416 |
| Number of income sources | 2.979 | 1.49 | 2.586 | 1.434 | 2.862 | 1.407 |
| Land size | 2.762 | 7.913 | 10.103 | 9.842 | 2.198 | 2.924 |
| Total livestock units | 14.467 | 19.111 | 8.972 | 15.564 | 1.73 | 4.966 |
| Asset index |  |  |  |  |  |  |
| Poorest | 0.28 | 0.449 | 0.229 | 0.421 | 0.203 | 0.402 |
| Poor | 0.173 | 0.378 | 0.189 | 0.392 | 0.188 | 0.391 |
| Average | 0.154 | 0.362 | 0.203 | 0.403 | 0.201 | 0.401 |
| Non-poor | 0.199 | 0.399 | 0.187 | 0.39 | 0.203 | 0.402 |
| Rich | 0.194 | 0.396 | 0.192 | 0.394 | 0.204 | 0.404 |
| Travel time to nearest town (minutes) | 79.939 | 80.118 | 51.06 | 34.795 | 70.223 | 50.561 |
| Log aridity | 8.145 | 0.209 | 7.826 | 0.082 | 8.765 | 0.429 |
| Human footprint | 11.153 | 4.35 | 6.131 | 3.384 | 8.697 | 5.076 |
| Respondent’s gender is female | 0.567 | 0.496 | 0.675 | 0.469 | 0.462 | 0.499 |
| N | 654 | | 428 | | 680 | |

Table A2 Association of COVID-19 with food security (indices) by country

|  | PCA Index | | |  |  | Standardized score | | |
| --- | --- | --- | --- | --- | --- | --- | --- | --- |
|  | (1) | (2) | (3) |  | (4) | (5) | (6) | (7) |
| VARIABLES | Kenya | Tanzania | Namibia |  | All countries | Kenya | Tanzania | Namibia |
|  |  |  |  |  |  |  |  |  |
| COVID-19 | 0.498** | 0.239* | 0.351 |  | 0.223*** | 0.301** | 0.113 | 0.235 |
|  | (0.193) | (0.136) | (0.225) |  | (0.080) | (0.149) | (0.090) | (0.142) |
| Household head is male | 0.088 | -0.080 | -0.008 |  | 0.035 | 0.074 | 0.006 | -0.019 |
|  | (0.108) | (0.160) | (0.144) |  | (0.057) | (0.088) | (0.111) | (0.099) |
| Secondary education | -0.044 | -0.007 | -0.355** |  | -0.101 | -0.075 | 0.000 | -0.273** |
|  | (0.102) | (0.198) | (0.171) |  | (0.062) | (0.082) | (0.134) | (0.107) |
| Age of the respondent | -0.001 | 0.006* | 0.000 |  | 0.001 | -0.001 | 0.001 | -0.001 |
|  | (0.003) | (0.004) | (0.005) |  | (0.002) | (0.003) | (0.002) | (0.003) |
| Household size | -0.008 | -0.061* | 0.039 |  | -0.011 | -0.003 | -0.036* | 0.024 |
|  | (0.014) | (0.033) | (0.024) |  | (0.009) | (0.012) | (0.021) | (0.016) |
| Number of rooms | 0.038 | 0.023 | -0.006 |  | 0.008 | 0.016 | 0.026 | -0.006 |
|  | (0.034) | (0.032) | (0.070) |  | (0.016) | (0.026) | (0.020) | (0.046) |
| Household access to electricity | -0.125 | 0.298** | -0.122 |  | 0.051 | -0.127** | 0.233*** | -0.095 |
|  | (0.076) | (0.113) | (0.212) |  | (0.050) | (0.058) | (0.077) | (0.135) |
| Household access to internet | -0.095 | -0.137 | -0.188 |  | -0.040 | -0.019 | -0.056 | -0.102 |
|  | (0.112) | (0.171) | (0.235) |  | (0.067) | (0.100) | (0.111) | (0.151) |
| Crops in storage | 0.162 | -0.094 | -0.429* |  | 0.058 | 0.411** | 0.072 | -0.283* |
|  | (0.213) | (0.124) | (0.231) |  | (0.078) | (0.185) | (0.088) | (0.145) |
| Locusts infestation | 0.112 | 0.146 | 0.416* |  | 0.110* | 0.107 | 0.099 | 0.331** |
|  | (0.116) | (0.135) | (0.231) |  | (0.056) | (0.085) | (0.091) | (0.148) |
| Job loss during COVID-19 | 0.102 | 0.048 | 0.098 |  | 0.143* | 0.198* | 0.104 | 0.076 |
|  | (0.110) | (0.194) | (0.187) |  | (0.080) | (0.102) | (0.121) | (0.115) |
| Membership in associations | 0.116* | -0.130** | 0.003 |  | -0.041 | 0.109** | -0.074** | 0.007 |
|  | (0.059) | (0.056) | (0.124) |  | (0.029) | (0.053) | (0.035) | (0.083) |
| Number of pre-COVID-19 shocks | -0.010 | 0.053 | -0.000 |  | 0.014 | 0.001 | 0.034 | 0.003 |
|  | (0.015) | (0.036) | (0.048) |  | (0.011) | (0.014) | (0.023) | (0.032) |
| Number of pre-COVID-19 shock responses | -0.008 | 0.021 | 0.181 |  | 0.042 | 0.005 | 0.009 | 0.111 |
|  | (0.047) | (0.149) | (0.149) |  | (0.045) | (0.038) | (0.103) | (0.099) |
| Number of income sources | 0.042 | -0.017 | -0.062 |  | 0.007 | 0.037* | -0.009 | -0.044 |
|  | (0.025) | (0.037) | (0.047) |  | (0.014) | (0.020) | (0.026) | (0.030) |
| Land size | -0.002 | -0.014 | -0.014** |  | -0.006* | -0.001 | -0.003 | -0.009** |
|  | (0.005) | (0.030) | (0.007) |  | (0.003) | (0.003) | (0.018) | (0.004) |
| Total livestock units | -0.004 | -0.027** | 0.008** |  | -0.003 | -0.003 | -0.020** | 0.005** |
|  | (0.003) | (0.013) | (0.004) |  | (0.002) | (0.002) | (0.009) | (0.002) |
| Asset index (base: rich) |  |  |  |  |  |  |  |  |
| Poorest | 0.262* | 0.244 | 0.078 |  | 0.046 | 0.200* | 0.201 | 0.077 |
|  | (0.151) | (0.240) | (0.209) |  | (0.076) | (0.117) | (0.167) | (0.138) |
| Poor | 0.214 | 0.512** | -0.028 |  | 0.099 | 0.182 | 0.344** | -0.001 |
|  | (0.199) | (0.221) | (0.237) |  | (0.089) | (0.143) | (0.153) | (0.160) |
| Average | 0.183 | 0.322 | 0.074 |  | 0.047 | 0.060 | 0.250* | 0.083 |
|  | (0.118) | (0.193) | (0.189) |  | (0.072) | (0.100) | (0.135) | (0.125) |
| Non-poor | 0.089 | 0.251 | -0.114 |  | -0.037 | 0.057 | 0.183 | -0.047 |
|  | (0.098) | (0.252) | (0.199) |  | (0.065) | (0.075) | (0.170) | (0.125) |
| Travel time to nearest town (minutes) | 0.000 | 0.003 | 0.004 |  | 0.001 | 0.000 | 0.002* | 0.002 |
|  | (0.002) | (0.002) | (0.004) |  | (0.001) | (0.001) | (0.001) | (0.002) |
| Log aridity | 0.819* | -0.855* | 9.140*** |  | -0.160 | 0.535* | -0.649** | 6.475*** |
|  | (0.432) | (0.485) | (2.648) |  | (0.275) | (0.317) | (0.317) | (1.725) |
| Human footprint | 0.020 | -0.040** | 0.001 |  | -0.003 | 0.018 | -0.024** | -0.001 |
|  | (0.016) | (0.017) | (0.024) |  | (0.010) | (0.014) | (0.011) | (0.015) |
| Respondent’s gender is female | 0.053 | 0.366** | -0.277 |  | 0.032 | 0.075 | 0.129 | -0.172 |
|  | (0.085) | (0.150) | (0.187) |  | (0.055) | (0.071) | (0.104) | (0.125) |
| Constant | -7.151* | 6.448 | -71.868*** |  |  | -4.535 | 4.957* | -50.920*** |
|  | (3.778) | (4.385) | (20.973) |  | 1.009 | (2.793) | (2.873) | (13.659) |
|  |  |  |  |  | (2.163) |  |  |  |
| Observations | 654 | 669 | 419 |  | 1,742 | 654 | 669 | 419 |
| R-squared | 0.157 | 0.146 | 0.153 |  | 0.285 | 0.221 | 0.126 | 0.169 |

Table A3: Probit regression results for the association of mobile phone ownership on attrition.

|  | **Attrited** | **Attrited** |
| --- | --- | --- |
| Has mobile phone | 1.1 | 1.17 |
|  | (0.93 – 1.30) | (0.95 – 1.43) |
| Was able to save money last month |  | 1.06 |
|  |  | (0.90 – 1.25) |
| Uses Mobile Money |  | 0.97 |
|  |  | (0.83 – 1.13) |
| Age of respondent in years |  | 1 |
|  |  | (1.00 – 1.01) |
| Has income from Business |  | 1.1 |
|  |  | (0.91 – 1.32) |
| Head has secondary education |  | 1.16 |
|  |  | (0.99 – 1.35) |
| Number of Associations |  | 0.94 |
|  |  | (0.85 – 1.05) |
| Total number of income sources |  | 0.97 |
|  |  | (0.93 – 1.03) |
| Household head is Male |  | 0.87 |
|  |  | (0.75 – 1.01) |
| Total number of shocks |  | 0.96 * |
|  |  | (0.93 – 1.00) |
| Average number of responses for past shocks |  | 1.14 |
|  |  | (0.98 – 1.31) |
| Land Owned in Ha |  | 1 |
|  |  | (1.00 – 1.01) |
| Tropical Livestock Unit |  | 1 |
|  |  | (1.00 – 1.01) |
| Number of household members |  | 0.98 |
|  |  | (0.96 – 1.01) |
| Constant | 0.32 *** | 0.36 *** |
|  | (0.28 – 0.38) | (0.25 – 0.52) |
| Observations | 2228 | 2110 |
| R2 Nagelkerke | 0.001 | 0.025 |
| AIC | 1861.265 | 1736.57 |

Significance levels corresponding with * p<0.1 for 10%, ** p<0.05 for 5% and *** p<0.01 for 1%
